# Yield and growth duration of Maroon rice landraces measured in traditional settings

**DOI:** 10.1007/s10722-024-02093-1

**Published:** 2024-07-30

**Authors:** Nicholaas M. Pinas, Jerry R. Tjoe Awie, R. Evanne Dongstra, Harro Maat, M. Eric Schranz, Marieke S. van de Loosdrecht, Tinde van Andel

**Affiliations:** 1 Naturalis Biodiversity Center, Leiden, The Netherlands; 2 Biosystematics Group, Wageningen University, Wageningen, The Netherlands; 3 Anne Van Dijk Rijst Onderzoekscentrum Nickerie (SNRI/ADRON), Nickerie, Suriname; 4 Knowledge, Technology & Innovation Group, Wageningen University, Wageningen, The Netherlands; 5 Clusius Chair in History of Botany and Gardens, Leiden University, Leiden, The Netherlands

**Keywords:** Maroons, Rice, Landraces, Suriname, Yield, Traditional agriculture, French Guiana

## Abstract

Rice, the most consumed cereal worldwide, has two domesticated species: Asian and African rice. Commercial cultivars, almost all Asian rice, are mostly selected for high yields. Traditional landraces are often selected for different traits, such as time to maturity or resistance against common stress factors, including bird attacks and poor soils. Maroons, descendants of enslaved Africans in Suriname and French Guiana, cultivate a rich diversity of rice landraces. They categorize landraces into short, medium, and long-maturation and maintain that fast-ripening crops with moderate yields facilitated their flight from enslavement, while once settled in a safe place, there was time for landraces with longer ripening periods and higher yields. Previous authors assumed that all Maroon landraces had low yields (700–1000 kg/ha), but their performance in traditional farming systems had never been investigated. We followed the growth and harvest of 28 Maroon rice landraces and two commercial cultivars in Maroon farmer fields, using traditional farming methods, at three locations. We show that, in farmer-managed fields, Maroon rice can yield 2600 kg/ha (average 1665 kg/ha), without any agrochemicals or machinery. Contrastingly, the commercial cultivars in Maroon farmer fields had a low yield (625–1205 kg/ha), partly due to bird predation. The maturation time varied between 110 and 183 days, but the three Maroon maturation categories showed significant overlap. Our study indicates that Maroon rice performance can only be fairly evaluated if measurements are taken in traditional Maroon farming systems, since this is the environment in which these landraces were originally selected.

**Supplementary Information:**

The online version contains supplementary material available at 10.1007/s10722-024-02093-1.

## Introduction

Rice is one of the most consumed staple food in the world and has two domesticated species: Asian rice (*Oryza sativa* L.) and African rice (*O. glaberrima* Steud.). Almost all commercial rice cultivars are Asian. African rice is grown in much smaller quantities in West Africa (Teeken et al. 2012), and in small patches in Maroon fields in Suriname, and French Guiana (Pinas et al. 2024). Due to a growing world population, the demand for Asian rice is projected to increase to 567 million tonnes in 2030 (Rahman and Zhang 2023). The variation in most rice fields is very low, since the so-called Green Revolution when a limited number of cultivars were promoted that responded well to mineral fertilizer application (Nguyen and Ferrero 2006; Nie and Peng 2017; Nori et al. 2008). Although much higher yields can be obtained with these cultivars, they appeared to be vulnerable to diseases, countered by spraying large amounts of pesticides. Regarding total quantity, rice is one of the crops on which most pesticide is used in India (Arora et al. 2019). A substantial share of the productivity increase was achieved by reducing the growth duration, improving irrigation infrastructure, and increasing acreage. This was only possible through labor-saving techniques. Machines for direct seeding and harvesting and herbicides for weed control are the main examples (Larsen and Noack 2021; Rao and Madhulety 2005).

Nevertheless, in many rice-growing regions manual transplanting, weeding, and harvesting are still common. Likewise, thousands of different rice cultivars are still grown by small-scale farmers in Asia, Africa, and the Americas for home consumption and small-scale trade (Li et al. 2014; Gopi and Manjula 2018; Stein et al. 2018). These traditional crop types are usually labeled as landraces, in contrast to modern, fertilizer-responsive rice cultivars, developed by breeding institutes. Landraces, developed by local farmers, have various advantages, in particular resistance against biotic and abiotic stresses, resulting in a stable, intermediate yield under low-input agricultural systems (Zeven 1998). Rice farmers know about these advantages and select particular combinations of rice cultivars or landraces to anticipate ecological differences and social needs. Despite policies of national governments and development organizations to stimulate commercial rice cultivation using modern, fertilizer-responsive cultivars, for many farmers landraces are a better option.

In Sierra Leone, for example, farmers in the 1900s selected *O. glaberrima* landraces named ‘pende’ that produced ripe seeds within 85–90 days (Hugh 1908; Richard 1986). In the 1980s, these landraces were still cultivated, and considered ‘hunger rice’, because they quickly produced a small amount of food when other rice cultivars were not yet ripe (Richards 1986). Farmers who could cope well with the situation during the Sierra Leone civil war (1991–2002) had mainly grown landraces capable of producing ripe seeds within 90 days, ensuring their communities in hiding had staple food within a short time (Richards 2006). Moreover, landraces are often selected on different merits than yield alone. In Sri Lanka, traditional rice farmers cultivate over 2400 landraces. One variety is grown specifically for breastfeeding women, another is for men going out to work in the fields, while local monks eat a variety with a high protein content (Dharmasena 2010).

In this paper, we focus on rice farming in Suriname, where the contrast between commercial rice cultivars and traditional landraces is very present. Commercial rice farming using modern, fertilizer-responsive cultivars is applied in the coastal zone, by farmers descending from Asian contract laborers brought to the colonial plantations in the late nineteenth and early twentieth century (Young and Angiers 2010). The use of landraces is very prominent among Maroon in the forested interior. Maroons are descendants of enslaved Africans who escaped the plantations in the late seventeenth and eighteenth centuries and established independent communities in the remote interior of Suriname and French Guiana (Price 1993). Maroons have continued their distinct rice farming activities, and grow landraces of both *O. sativa* and *O. glaberrima* (van Andel et al. 2019). Rice is a keystone crop in all Maroon communities: it has been their staple food for centuries, enabling them to survive independently from coastal societies (Pinas et al. 2023; van Andel et al. 2019). The Dutch colonial government started growing rice as bulk food for enslaved Africans and soldiers in 1688 (Elfrink et al. 2024). Expeditions sent out to capture Maroons mentioned the first provision of fields with rice in 1712 (Dragtenstein 2002). In the Cottica area, where large groups of runaways hid in the swamps behind the plantations at the end of the eighteenth century, huge rice fields were discovered by the armies sent to trace them (Stedman 1988). These large rice fields suggested that runaways depended on rice for their survival.

Maroon provision fields are radically different from commercial rice fields in the coastal area of Suriname. Commercial rice fields are established in open, wet polders on clay soils, with semi-mechanical sowing and harvesting (Mitro 2010; Ten Have 1967). Commercial rice is grown as a monoculture crop, for which the use of fertilizers, pesticides, and herbicides is a must. Contrastingly, Maroons grow multiple crops, such as cassava, okra, plantain, sugarcane, and rice in one field. Trees are felled to open a field in the forest and are burned rather than removed, and as they decay over time, they provide nutrition to the crops. Farmers do not apply mineral fertilizers and hardly ever use other agrochemicals.

Maroons cultivate many morphologically and genetically different rice landraces (Van Andel et al. 2019; Van de Loosdrecht et al. 2024) with several landraces sown in succession to even out labor during the harvest season (Pinas et al. 2023). It was suggested that during marronnage, short-ripening crops were favorable, as people could travel further once they had a little food (Pinas et al. 2024; Van Andel 2010). Once settled in a safe place there was adequate time for landraces that required a longer ripening but produced higher yields. In addition, a subset of the Maroon rice landraces has long awns. Awns have been proposed to contribute to a higher harvest yield by protecting against bird predation (Bullard 1988; Furuta et al. 2015; Portères 1966) and enable more photosynthesis close to the developing grain to increase the starch storage (Grundbacher 1963). Commercial cultivars generally have no awns because this trait is problematic for mechanical seed processing (Hua et al. 2015).

Maroon agriculture has been portrayed as primitive and unproductive in colonial and post-colonial times (Lobach 2023). Geijskes (1955) classified their shifting cultivation system as ‘wasteful land use’ and ‘primitive’ and considered the Maroons incapable of improving their agricultural methods themselves. Budelman and Ketelaar (1974) proposed that their dryland rice landraces should be replaced with modern wetland rice cultivars that were more profitable for trade and export. Nascente and Kromocardi (2017) and Sewnarain (2021) stated that low grain yields are problematic for the Maroons, as they rely on rice as their staple food. As yields were considered insufficient to meet the demand, they advised replacing the Maroon landraces with modern Brazilian upland cultivars to increase grain yield and meet food security needs. Several of the negative descriptions on Maroon rice production are sustained with figures about an estimated yield between 700 and 1000 kg per hectare (Budelman and Ketelaar 1974; Nascente and Kromokardi 2017; Young and Angier 2010). None of these investigations mentioned how they came to these yield estimates, and they have measured Maroon rice yield only in experimental settings.

In this paper, we present results from measurements of growth duration and yield of Maroon rice landraces, compared with two commercial cultivars from the coastal region of Suriname, in established fields of Maroon farmers who grow rice under rain-fed conditions without the use of irrigation, pest control, fertilizers, or herbicides. Maroons group rice landraces for ripening duration into short-, medium, and long-duration to even out labor intensity. However, Maroon farmers do not register exact sowing and harvesting dates, but their techniques have not been described. Hence, we aimed to describe sowing and harvesting techniques, and measure the maturation time of the Maroon rice landraces to confirm the maturation-duration classes assigned by the Maroon farmers and compare their yields. Lastly, we aimed to investigate whether the yield is higher for awned compared to non-awned rice cultivars of the same Maroon maturation class.

In our study, we address the following research questions:

1. What is the time to maturation of traditional landraces that the Maroons classify as short, medium, or long-duration rice?
2. What is the general yield of these landraces in the traditional farming system?
3. What is the time to maturation and yield of commercial Surinamese wetland cultivars under Maroon farming systems?

We hypothesized that short-duration landraces, favored during marronnage since it allowed the Maroons to travel further with food sooner (Pinas et al. 2024; Van Andel 2010), had low yields. Similar to West African farming systems (Richards 1986) and following Maroon’s oral history on marronnage, this ‘hunger rice’ could bridge the waiting time for the long-duration landraces to become ripe, which we expected to have higher yields. We also expected awned landraces to have higher yields because awns protect against bird predation (Furuta et al. 2015; Grundbacher 1963) and may enhance photosynthesis. As cultivars will produce best in the ecological environment for which they were selected and developed, we expected that commercial wetland cultivars would probably produce less yield in a Maroon farming system, because they were not bred for this environment without the input of agrochemicals.

## Material and methods

### Selection of landraces

**Fig. 1:**
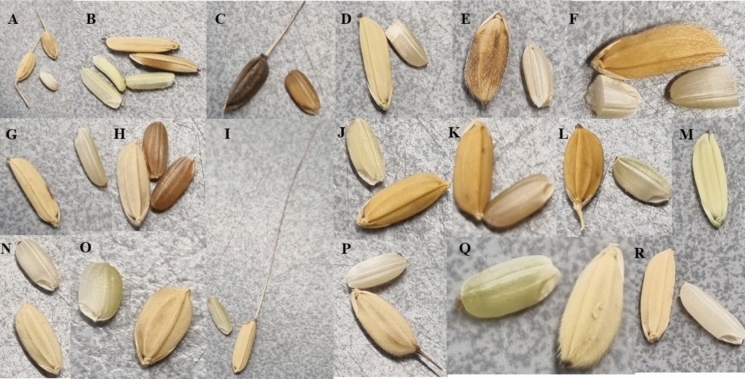
The distinct morphology of Maroon rice landraces harvested in this study. Individual photos are unscaled. **A** weti hédi, **B** wataa alisi, **C** mátu alisi, **D** baáka gogo, **E** ahunjön, **F** kwili kwili, **G** alëkisóóla, **H** jengejenge, **I** djampö, **J** fini sii, **K** bë alëkisóóla, **L** alëkisóóla baaw ana, **M** A125, **N** alisi seéi, **O** bë sika sii, **P** taanga lobi, **Q** weti sika sii, **R** weti kuli. All landraces are O. sativa, except 1C (O. glaberrima). Photographs: N. Pinas

In 2021 and 2022 we interviewed 67 Maroon farmers, collected over 400 rice samples, and documented information on their agronomic traits (Maat et al. 2023; Pinas et al. 2023). Based on this information, we selected a total of 28 landraces that the Maroon farmers had in stock and which they indicated either as short-duration (10, ready to be harvested between 3 to 4 months), 8 medium-duration (between 4 to 5 months), and 10 long-duration (between 5 to 8 months) (Suppl. Table 1). The 10 short-duration landraces for our study were mátu alisi, alisi seéi, alisi seéi bë wojo, jengejenge, kwili kwili, akwandjaa, bë sika sii, weti sika sii, weti alisi, and awéi máu (Fig. 1). The awned mátu alisi (or baaka alisi, Fig. 1C) is the only *O.glaberrima* that was collected in Suriname thus far (van Andel et al. 2016). For Maroons, this black-husked rice is of spiritual significance: its short growth season helped their ancestors to quickly produce food during marronnage (Pinas et al. 2024). All other short-duration rice was awnless. Alisi seéi is an *O. sativa* with white, hairy husks (Fig. 1N) that was named after a Ghanaian enslaved woman who escaped slavery around 1690 (Van Andel et al. 2023).

Four of the eight landraces that we selected and that were classified by the Maroons as medium-duration had extremely long awns (weti hédi, fini sii, djampö, and sika sii ku puma, see Fig. 1I). We selected them to see whether awned rice had a higher yield than awnless rice. The other four (awnless) medium-duration landraces were bë kuli, weti kuli, and two types of ahunjön. Ahunjön is a Maroon synonym of the term ‘pende’: an *O. sativa* landrace with spots on the husk (Fig. 1E). In the Mende language (Sierra Leone), pende means ‘dark’ (Hugh 1908) and this name is given to *O. glaberrima* that ripen very quickly (Richards 1986).

The 10 long-duration landraces that we selected, all awnless, were masaa alisi, wataa alisi, alëkisóóla, bë alëkisóóla, alëkisóóla baaw ana, weti alëkisóóla, taanga lobi, katam, baaka gogo and bambusi. Alëkisóóla is a popular *O. sativa* variety in Maroon communities with an elongated seed and white husk (Fig. 1G). It is related to the USA cultivar Rexora, imported to Suriname in the 1930s and extensively experimented with in the coastal fields (Stahel 1944; Baumgart et al. 1998; Van de Loosdrecht et al. 2024). During our interviews, Maroons often reported that alëkisóóla had a longer ripening period: it is sown as one of the first and harvested as one of the last. Farmers indicated it as a high-yield rice. Masaa alisi is an *O. sativa* landrace cultivated only in the swamps of the Cottica region. Farmers indicated that it was ready for harvest after six to seven months, which would be the longest for all Maroon rice we have collected thus far. It is grown only in deep swamps and is said to be able to compete with grass. It is a very tall variety with elongated seeds, brown hairy husks, and a mix of red and white bran. Wataa alisi is a landrace with elongated seeds (Fig. 1B) that was sown in an area of the field that holds water, as indicated by the farmer. Two other landraces grown in swamps (katam and bambusi) were also indicated as long-duration swamp rice by the Cottica farmer. In addition, two commercial wetland cultivars (A-125 and A-130) developed by the Surinamese Rice Breeding Institute (SNRI/ADRON), of which detailed agronomic information was known, including yield per hectare (Tjoe Awie 2010) were handed out to two farmers for sowing as well.

We collected seed samples and whole rice plants for herbarium vouchers for all them that came to full maturation during our fieldwork. One duplicate was deposited at the National Herbarium of Suriname (BBS) in Paramaribo, Suriname, and the other at the Herbarium of Naturalis Biodiversity Center (L) in Leiden, the Netherlands. Living seeds of each were deposited at the SNRI/ADRON germplasm bank in Nickerie, Suriname for storage, phenotyping, and multiplication. Duplicates grown by SNRI/ADRON were later deposited at the Svalbard Global Seed Vault, Norway.

### Choice of locations

**Fig. 2:**
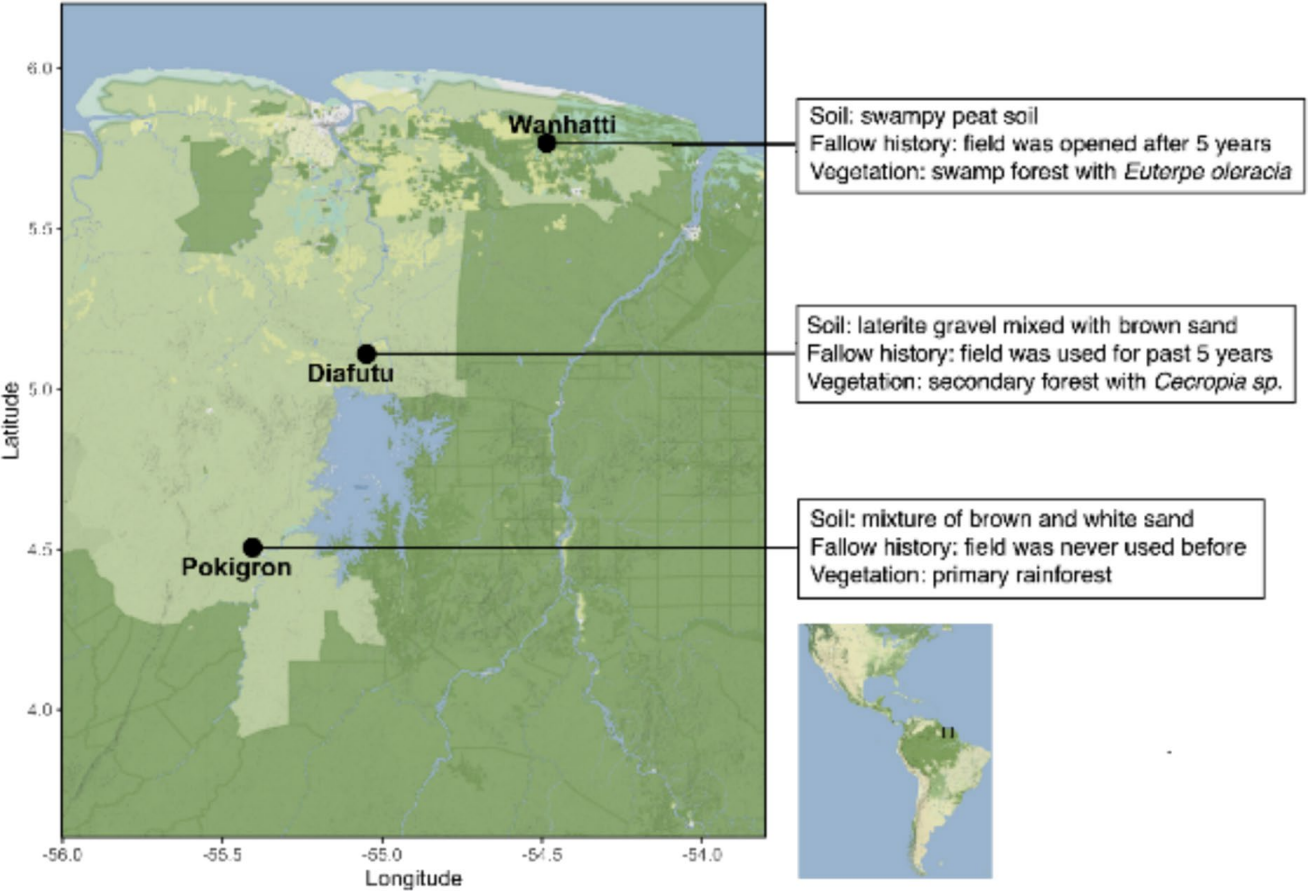
Field locations with agroecological characteristics where Maroon rice performance measurements were conducted

We selected locations that were easy to reach by car, represented at least two of the major Maroon groups (Saamaka and Okanisi), and also the different soil types on which the Maroon rice is typically grown. Wanhatti is an Okanisi village in the swampy Cottica region, Pokigron is a Saamaka village located in the Sipaliwini district with sandy soil and Diafutu is a Saamaka village near Brokopondo city center (Fig. 2). In December 2022, we selected farmers who planned to sow the rice landraces and cultivars we wanted to study. We already had interviewed these farmers in 2021 and/or 2022 and they confirmed their willingness to participate. Farmers were compensated with on average 20 USD per fieldwork day (500 SRD). The farmers were Mrs. Julia Pomba (Saamaka, Diafutu); Mrs. Anne Huur (Saamaka, Pokigron), and Mrs. Marjorie Kanawi (Okanisi, Wanhatti).

The husbands or male relatives of the farmers did the field preparation, such as clearing and burning, on their own initiative. Preparing the soil and sowing by hand broadcasting was done by the (female) farmers. We regularly contacted them via WhatsApp to be informed of their activities. Sowing was done in April; the farmers determined and recorded the date. We demarcated three plots of 0.25 square meters with four wooden sticks of 50 cm each forming a square. The location of each variety was marked in the field with a wooden board on a stick and the collection number (e.g., NP 405) of the landrace was written on it with black paint. We counted the number of seeds sown per 0.25 square meter for several landraces and cultivars, and an average was calculated for each farmer.

Three plots of 0.25 square meters were randomly arranged within a part of the field with the same rice variety. Yields may differ within a gradient because of potential differences in organic material in the soil (Gomez 1972). Therefore, if there were height differences within the patch where one variety was sown, we placed a plot on the highest point, one at the middle, and one at the lowest.

### Measurements

Measurements for sowing density, plant height and grain yield were done in July–August 2023. Harvesting started when the farmer indicated that the rice was ripe.

*Sowing density:* We determined the average sowing density for each farmer by counting the number of seeds sown in each of the three plots and determined an average across all plots and landraces or cultivars.

*Plant height*: Plant height was measured as the distance in cm between the soil surface and the height of the tallest panicle on plants when the crop was ripe (IRRI 2013). For each rice variety, 10 plants were randomly selected for plant height measurements within each plot to determine average height (Table S1.A: average plant height [cm]). Based on their average height at maturity, Maroon rice landraces were categorized as semidwarf (< 90 cm), intermediate (90–125 cm), and tall (> 125 cm), based on the categories for upland rice landraces (IRRI 2013). Commercial cultivars typically fall into the categories semidwarf (< 110 cm), intermediate (110–130 cm), and tall (> 130 cm), categories that differ from those of landraces (IRRI 2013).

*Grain yield:* Grain production was measured by hand after the grain reached physiological maturity in each plot. We determined the grain yield of rice by measuring three components:

1. Ten plants were randomly selected for tillers counting within each plot (IRRI 2013).
2. Number of tillers per plot. This was determined by counting all the tillers in the plots and averaging the data (Table S1.A: average # tillers per plot [0.25 m^2^]). The conventional perspective is that high-tillering rice plants have a higher yield per individual plant (Mohanan and Pavithran 2007). High tiller-producing cultivars can have between 20 and 25 tillers, of which approximately 10 will be unproductive (Pawar et al. 2016). As such, the number of productive and nonproductive tillers of each variety was counted (Table S1.A: average # productive tillers per plot [0.25 m^2^] and average # non-productive tillers per plot [0.25 m^2^]).
3. Numbers of fully developed and underdeveloped grains per panicle per plot (Table S1.A: average # productive grains per panicle per plot [0.25 m^2^] and non-productive grains/panicle per plot [0.25 m^2^]). This was determined by counting the number of grains in 10 panicles, randomly sampled in one plot, dividing by 10 (Gomez 1972) and the average taken over all three plots.
4. 1000-grain weight. For each variety, all rice panicles were collected from the three plots. The bundles of rice panicles were labeled and transported in bags to the SNRI/ADRON lab facilities. There the unfilled grains from the rice were removed by hand, and the husks of fully developed grains were removed in an electric rice mill. In addition, moisture content was reduced to 14% (IRRI 2013) with a rice moisture dryer (Memmert, UF750). We determined the 1000-grain weight for the milled rice, 14% moisture content corrected grain by measuring 100 grains taken randomly from each plot, averaging over the three plots, and multiplying the average weight by 10 (Table S1.A: 1000 grains weight [gr] at 14% water content).
5. Total yield. The total yield was determined by weighing the (milled) grain samples from the three sampling plots from each variety and corrected to 14% moisture (Table S1.A: average milled grain weight 14% moisture [gr/0.25 m^2^]). This average yield of the three sampling plots was multiplied by 40,000 to reach an average yield in kg per hectare (Table S1.A: average yield [kg/ha]).

### Data analyses and statistics

Data analysis was done in Rstudio with Base-R version 4.3.2. For a total of 24 rice landraces all performance variables could be measured (Pokigron: n = 16, Diafutu: n = 6, Wanhatti: n = 2). Given this modest sampling size we first tested the normality of the data for each of the measurement variables using the Shapiro–Wilk Test (*Shapiro. test()* function, stats package) and made Q-Q plots (*qqPlot()* function, car package) (Table S1.B). We found the data for most variables to have a non-normal distribution (seed density, maturity time, plant height, and total yield).

Each variable was subsequently investigated using nonparametric tests for the effects of the Maroon maturation class (Table S1.C). When a significant effect was detected with a Kruskall-Wallis Test (*Kruskal. test()* function, stats package), we subsequently applied the Dunn’s Test with a Bonferroni correction (*dunnTest()* function, FSA package) to test the medians for all pairwise combinations of the maturation or location groups. We caution that the sample group sizes are asymmetric and (very) small during the statistical testing for differences in yield performance between maturation classes (4 < n < 8) and locality (2 < n < 16), and as such may affect the reliability of these tests. In addition, with a Mann–Whitney U Test (Wilcox*. test()* function with ‘exact = FALSE’, stats package) we investigated whether the Maroon landraces as a combined group (n = 16) differed from the commercial cultivars (n = 4) for each of the performance variables measured (Table S1.D).

*Seed density correction for relevant yield measurements.* Differences in sowing density may drive differences in observed yield. Therefore, we applied a correction factor to minimize any effects on yield due to differences in sowing density. The correction factor is proportional to the relative difference in sowing density to the site with the lowest sowing density (Diafutu). As such, the correction factors for all grain production measurements are Daifutu: 1.00, Pokigron: 0.86, and Wanhatti: 0.70. This correction factor was applied to the ‘Number of fully developed grains per panicle per plot’, ‘Average milled grain weight with 14% water content’ and ‘Total yield’ variables.

## Results

### Sowing

From March until mid-April 2023, farmers were busy cleaning, burning, and preparing their fields. Sowing started on April 17th, just after it started to rain, and continued until April 25th. The Maroon farmers did not consult the weather forecast for updates on rainfall, but instead listened to a local cicada named siksi-yuru** (Fidicina mannifera** Fabricius 1803). This insect had to stop ‘singing’ his loud song, which indicated that it would soon start to rain. We found that farmers made no difference in sowing density between their landraces, but densities differed per farmer. In Pokigron each plot had an average of 92 seeds, in Diafutu 79 seeds, and in Wanhatti 112 seeds.

### Harvesting

In July, August, and September the first author harvested 20 Maroon landraces and two commercial cultivars in the three locations. Masaa alisi was harvested in November, outside the fieldwork period. The landraces katam, bambusi, and one type of ahunjön did not sprout: the farmer indicated these were old seeds that could not germinate anymore. Akwandjaa, weti alëkisóóla, awéimáu and sika sii ku puuma germinated, but only reached average heights between 10 and 15 cm. According to the farmer, this happened because the patch of field where they were sown had no charcoal or other burned plant material to nourish the rice plants.

### Plant height

**Fig. 3:**
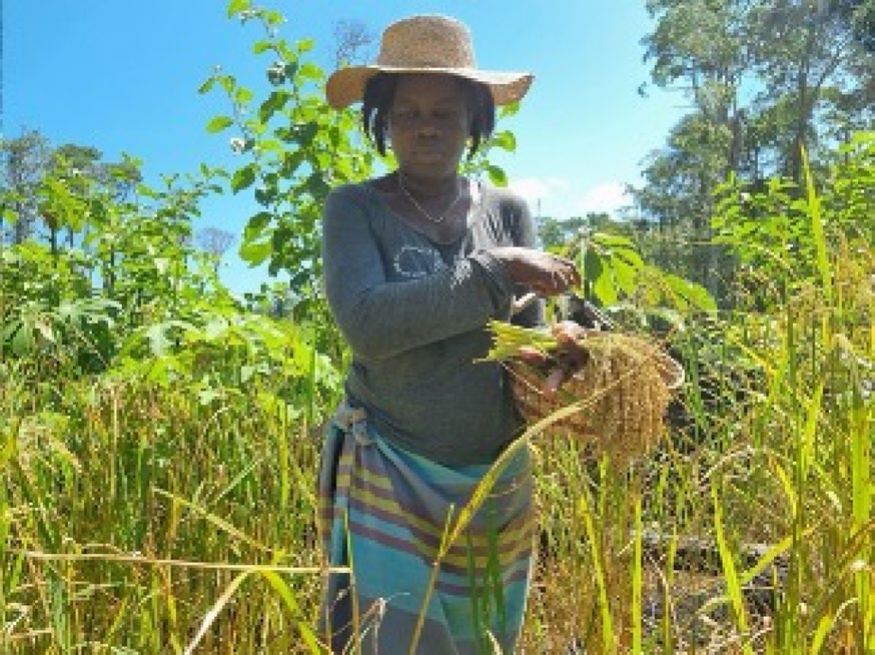
(A) Mrs. Anna Huur harvesting inside a patch of Maroon rice in Pokigron. The panicles grow at the level of her hands, facilitating the harvesting process. Photo: N. Pinas

Maroons have developed their landraces to a height at which panicles are at the level of the farmer’s hands (Fig. 3). In this way, panicles can be cut while standing upright, which is experienced as less labor-intensive than bending down. Indeed, we found that all the Maroon landraces were taller than 90 cm at maturity (Table S1.A). The variety weti hédi had an average height of 110 cm and fell in the intermediate category for upland cultivars (IRRI 2013). The remaining 19 landraces fell within the tall category with average heights of 132.7–177.6 cm (median 160.0 cm) with wataa alisi (177.6 cm) and alisi seéi bë wojo (176.9 cm) being the tallest.

With average heights of 43.3–60.0 cm (median: 56.6 cm), the two commercial cultivars A-125 and A-130 were categorized as semidwarf (< 110 cm for lowland cultivars) (IRRI 2013). Notably, their plant height was half the height they reached when grown in commercial rice fields in Nickerie (on average 105 cm) (Tjoe Awie 2004). In addition, we observed a difference in tillers between the Maroon landraces and the commercial cultivars. The median in the 28 Maroon landraces was 1.2 tillers, whereas for the commercial cultivars A-125 and A-130 the median was 4.0 tillers. This higher number of tillers is typical for commercial cultivars (Mohanan and Pavithran 2007).

### Days to maturity

**Fig. 4:**
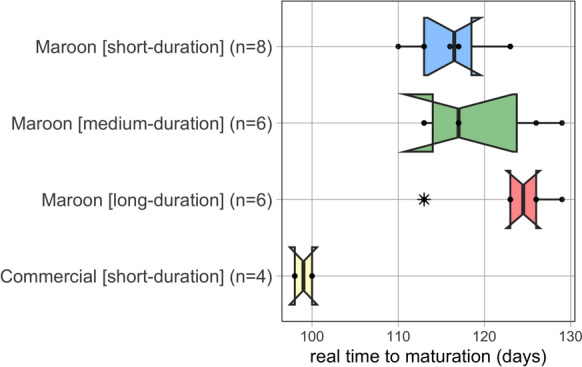
Box plots for the different maturation classes as indicated by Maroon farmers. Every dot represents a Maroon rice landrace. The landrace masaa alisi (183 days) is not shown. The maturation times for the three Maroon landrace categories overlap. See Supplementary Table 2

The median maturation time for the Maroon landraces was 117 days (slightly less than 4 months). The fastest ripening Maroon rice was mátu alisi (*O. glaberrima*) at 110 days (~ 3.5 months) (Table S1.A). The landraces alëkisóóla and weti hédi both took 129 days (~ 4 months) to mature, although Maroons classify alëkisóóla as a long-duration variety and weti hedi as a medium-duration variety. The commercial cultivars, however, were mature between 98 and 100 days (just over 3 months), similar to their phenology data from the SNRI/ Adron (Tjoe Awie 2010). The difference between the earliest maturing (mátu alisi) and most of the long-duration landraces was a maximum of 19 days. The exception was masaa alisi, indicated by Maroons as a long-duration variety, which was harvested at 183 days (more than 4.5 months). Unfortunately, the two other long-duration types, katam and bambusi, did not germinate due to bad seed quality, so we could not verify their maturation time. Notably, none of the Maroon landraces matured within three months, even though this was mentioned to us previously by farmers. Although Maroon farmers classify their rice into short-, medium-, and long-duration ripening, the maturation time for these three categories showed significant overlap (Fig. 4) and we did not detect statistically significant differences (Kruskal–Wallis Test: *X*^2^ = 4.069, *p* = 0.131 (Table S1.C). However, the maturation time of the commercial cultivars tended to be shorter than that of the Maroon landraces as a group (Mann–Whitney-U Test: *p* = 0.002, Table S1.D).

### Grain weight

**Fig. 5:**
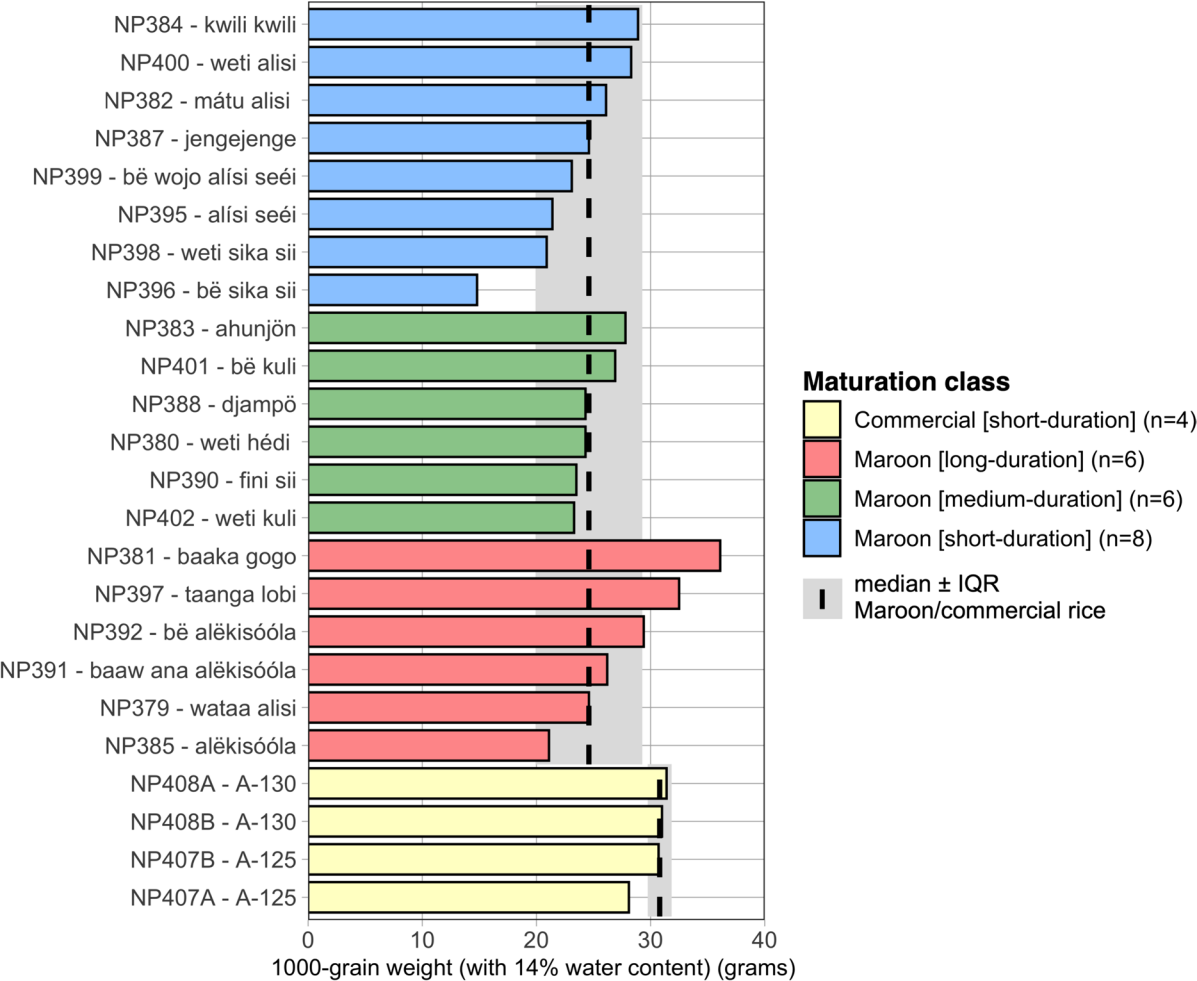
1000-grain weight at 14% water content of the 20 Maroon rice landraces. Colors reflect the maturation classes distinguished by the Maroons. The dotted line and grey area mark the median and interquartile (IQR) range for either all the Maroon landraces or the commercial cultivars as a group. Within each maturation class, there is variation in grain weight. See supplementary Table 2

The Maroon landraces had a median 1000-grain weight of 24.6 gr (Fig. 5). The landraces bë sika sii and weti sika sii had the lowest 1000-grain weight due to their tiny seeds (4–5 × 3–4 mm). Some of the ‘alëkisóóla’ types (baaka gogo, alëkisóóla, alëkisóóla baaw ana, and bë alëkisóóla) were among those that had relatively the highest grain weights because these thin, elongated rice types were relatively large (9–11 × 3–4 mm) (Fig. 5, Table S1.A). Overall, we did not detect a significant difference in grain weight between the maturation classes assigned by the Maroons (Kruskal–Wallis Test: *X*^2^3.054, *p* = 0.217) (Table S1.C). The commercial cultivars A-125 and A-130 had a 1000-grain weight of 30.8 gr on Maroon fields (Fig. 5), which appeared to be lower than the average 31.5 gr measured for the same landraces when planted in a commercial field (Tjoe Awie 2010).

### Total yield of landraces and commercial cultivars on Maroon farms

**Fig. 6:**
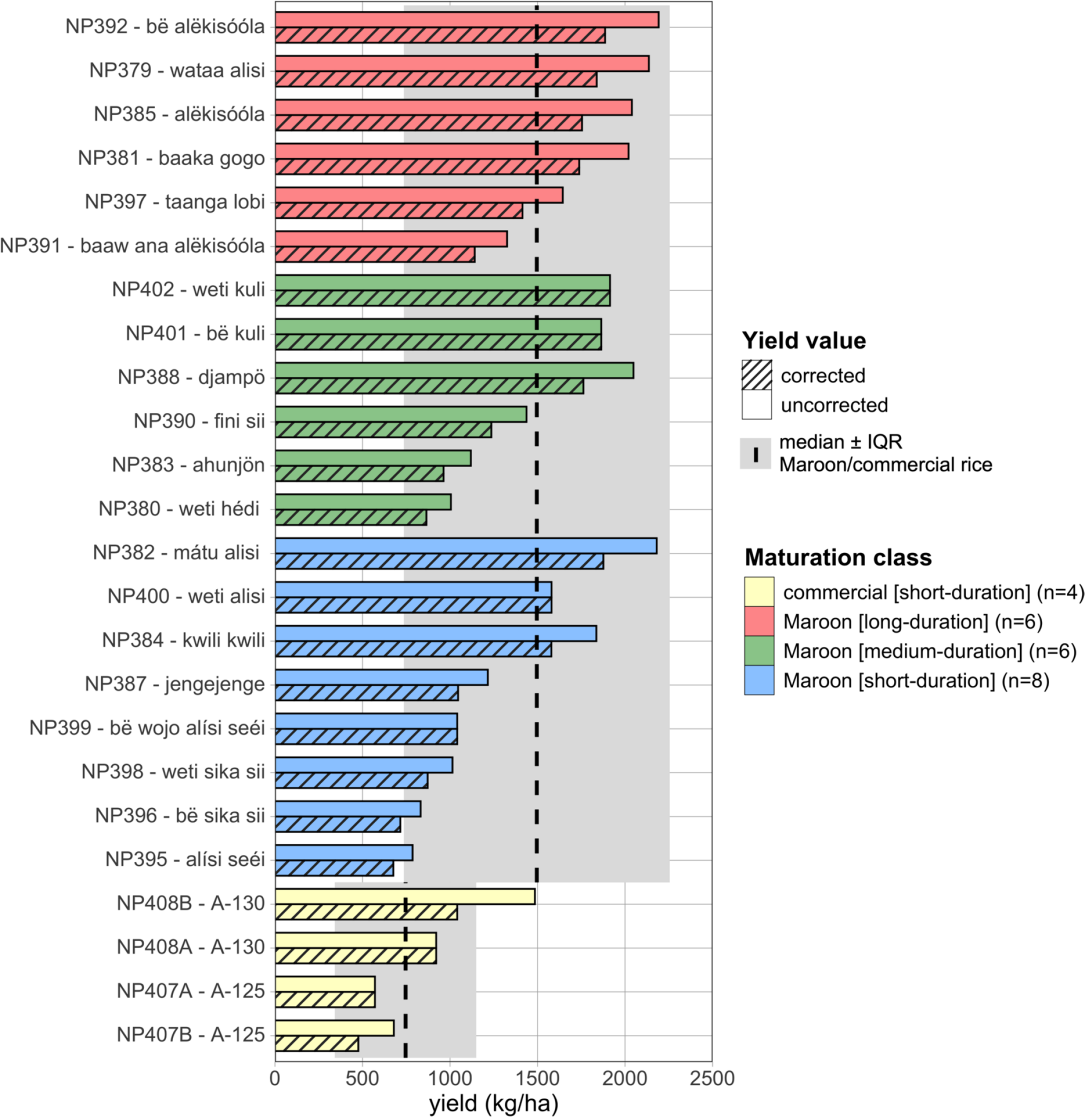
Yield in kilogram per hectare (kg/ha) with and without seed density correction. Colors reflect the maturation classes distinguished by the Maroons. The dotted line and grey area mark the median and interquartile (IQR) range based on the seed density corrected yield for either all the Maroon landraces or commercial cultivars as a group. The figure shows that there is no correlation between yield and maturation time. See supplementary Table 2

The Maroon landraces had a median (seed density corrected) yield of 1496 kg/ha (IQR: 736–2255 kg/ha) (Fig. 6). We did not detect a significant difference in yield between the Maroon maturation classes (Kruskal–Wallis Test: *X*^2^ = 3.433, *p* = 0.180) (Table S1.C). The Maroon landraces known as sika sii had relatively low yields (max. 872 kg/ha), which was expected due to their small seeds (Fig. 6). The reason for the low yield for alisi seéi (676 kg/ha) was unclear, as it had seeds of average length (8 × 5 mm). The Maroon landraces weti kuli, bë kuli, wataa alisi, mátu alisi, and bë alëkisóóla had yields above 1800 kg/ha. Congruently, the farmers we interviewed all indicated that the alëkisóóla types always produced a good yield.

**Fig. 7:**
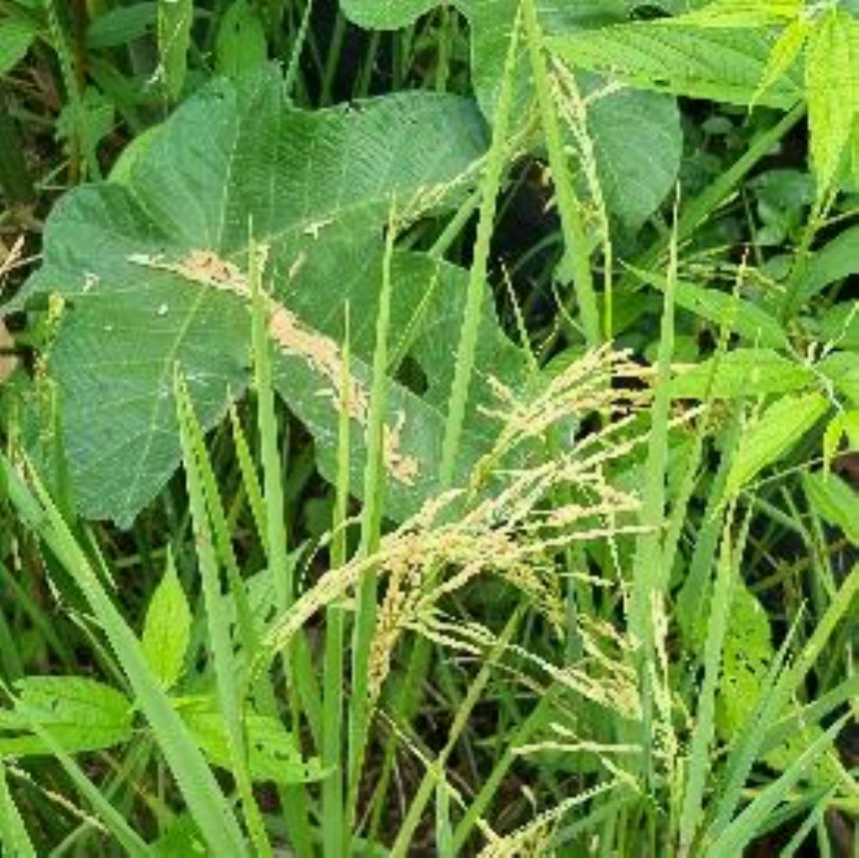
Commercial cultivar A-125 in the Maroon field at Diafutu showing signs of heavy bird predation, 1 August 2023. Empty rice husks are visible on a leaf of a *Cecropia* sapling. Photograph: N. Pinas

In industrial rice fields in Nickerie, the two commercial cultivars A-125, and A-130 can reach yields between 6100 and 6300 kg/ha (Tjoe Awie 2010). In Maroon fields, however, these two commercial cultivars produced much lower yields than in the industrial settings. A-125 and A-130 had maximum yields of only 571 and 1041 kg/ha, respectively (Fig. 5, Table S1.A). This low yield on Maroon fields is probably explained by the fact that Maroon farmers did not use agrochemicals. These modern cultivars were developed to have a strong response to artificial fertilizers and not to the natural source of minerals available on Maroon rice fields, such as charcoal and decaying plant material. Another plausible reason for the low yield of A-125 and A-130 was bird predation. The birds in Diafutu feasted on both cultivars (Fig. 6), while the Maroon landraces a few meters away in the same field were mostly left untouched (Fig. 7).

## Discussion

### Maturation time of Maroon rice

We did not see a clear difference between short- and medium-duration landraces in our study, even though Maroon farmers classified them as such. The approximately three weeks between early and late ripening of Maroon landraces seemed more important for the management of workload during harvest, even when all were sown at the same time. Two farmers said they sowed the distinct landraces on different days to spread the harvesting work more evenly. Other Maroon landraces with shorter ripening times may exist, but because of time constraints, we focused on only 30 of the hundreds of different Maroon landraces. When growth periods of all these would be measured in the environment in which they were selected, a clearer difference in maturation time may appear.

In previous experiments in Nickerie by Sewnarain (2021), the Maroon landraces took between 159 and 194 days to mature, much longer than in our study. Sewnarain also included Maroon swamp rice (‘masra’) in her experiments, which is probably the same variety as the long-duration masaa alisi in our study. In Nickerie, this swamp rice took 159–181 days to ripen, somewhat shorter than in our study (183 days). However, some of the awned Maroon rice that Sewnarain planted (ajojo and botomboli) had very long maturation times (181 to 194 days). This was probably caused by the heavy clay soils in Nickerie, which are absent in the Maroon communities.

### Yield of Maroon rice

The Maroon rice landraces we studied had a median yield of 1496 kg/ha (ranging between 760 and 2255 kg/ha) when corrected for sowing density, without agrochemicals or heavy machinery. This is higher than the previously assumed low yields of 700–1000 kg/ha (Budelman and Ketelaar 1974; Sewnarain 2021). Maroon rice yields today are comparable to the yield obtained by the first Asian contract laborers in Suriname, who harvested 1200–1500 kg/ha, and in the most favorable circumstances 2000 kg/ha, also without agrochemicals, in the coastal wetlands (Benjamins and Snelleman 1914–1917). These mid-19th-century yield data are the only figures available for traditional rice cultivation in Suriname. Rao et al. (2018) measured yields of rice landraces under low nitrogen conditions in India and distinguished high yielders (producing more than 3000 kg/ha in the wet season and more than 2500 kg/ha in the dry season), moderate yielders (> 1500 kg/ha), and low yielders (< 1500 kg/ha). According to those categories, 10 Maroon landraces can be considered as moderate yielders and 10 as low yielders.

We cannot rule out that the locations where we followed and harvested the landraces influenced the maturation time and yield. Although we did not measure environmental components, the same landraces may respond differently to other locations because of variations in rainfall, sun intensity, soil composition and structure, sowing density, availability of charcoal, and farmer practices. It is probable that such location-specific factors also influenced the outcomes of previous experimental research with Maroon rice. In the discussion about traditional crop landraces and yield, it should be taken into consideration under which conditions they were originally selected and developed. If yields and maturation periods are measured under experimental settings outside their traditional environment and without the farming practices under which they were developed, the results should be interpreted with care.

### No ‘hunger rice’ landraces in our selection

Our hypothesis that short-duration Maroon landraces would produce lower yields than long-duration ones was not confirmed by our data. Early-ripening landraces such as mátu alisi (110 days) had a corrected yield of 1876 kg/ha and kwili kwili (116 days) with 1579 kg/ha. While the alëkisóóla types generally took longer to ripen (up to 129 days) and generally produced more than 1700 kg/ha, alëkisóóla baaw ana (123 days) produced only ~ 1150 kg/ha. The landrace bë alëkisóóla was considered a long-duration type but took only 113 days to ripen and produced more than 1850 kg/ha. The difference in growth periods and yields within the ‘alëkisóóla-type’ landraces indicate that they are not as similar as their name suggests. We, therefore, reject our hypothesis that short-duration landraces’ yields are always lower and long-duration landraces are always higher. This contrasts with the Sierra Leone system as described by Richards (2006), in which ‘hunger rice’ (named pende) had a low yield but ripened within 90 days. The Maroon rice with the same name (ahunjön or pende) had a moderate yield (963 kg/ha) and took 126 days to ripen, much longer than its West African namesake (90 days). As such, the name retention seemed to refer to the spotted husk rather than its growth duration.

### Measuring Maroon rice production

Previous research suggested that Maroon landraces should be replaced with modern rice cultivars capable of producing higher yields (Nascente and Kromokardi 2017; Power 2015). Our results show that estimating Maroon rice yield depends on the selected landraces: small-seeded rice will generally produce lower yields than landraces with bigger seeds. Maroons generally sow fewer seeds per square meter (c. 375 seeds/m^2^) than farmers in commercial settings (c. 500 seeds/m^2^), so yields can only be compared when adjusted for sowing density. Under experimental settings, Nascente and Kromokardi (2017) measured an optimal yield for the Maroon rice landrace topi-topi of 1893 to 2429 kg/ha without agrochemicals. However, as they did not provide data on sowing density, comparing their yield with our data is difficult.

Maroons do not measure their production in kilograms per hectare but generally estimate how long a family can consume their homegrown rice. We interviewed farmers who had sufficient rice for a year on a relatively small field (c. 3 ha) with multiple crops besides rice. We also spoke with farmers who indicated they would consume their harvest within a week, as they only cultivated rice for traditional funeral ceremonies.

### Awned versus awnless landraces

Farmers informed us that when birds could choose between glabrous and hairy or awned rice, they would feed on the glabrous types. However, our data does not show differences in yield between landraces with awns (e.g., mátu alisi and djampö), hairy husks (wataa alisi), and glabrous and awnless landraces (e.g., alëkisóóla baaw ana). Based on field observations of the first author and Maroon farmers, only the commercial cultivars (A-125 and A-130), which were completely glabrous and awnless, suffered enormously from bird predation. This was especially true for Diafutu, where the rice field was located next to an agricultural plot that had been used for several years. In Wanhatti, the rice field was established in a swamp not recently used for planting, and the commercial cultivars were somewhat less attacked. ‘The birds are not yet aware that we made a new field there’, the farmer explained. Apart from awns and hairy husks, other traits of Maroon rice plants may play a role in preventing bird predation.

In a previous experiment from the SNRI/ADRON with the glabrous and awnless cultivars A-125, A-127, A-128, and A-130 in Victoria (Brokopondo district) there was no harvest at all, due to heavy bird predation and disease (Tjoe Awie 2013). The sowing date (July 17th) was also outside the sowing period used by Maroon farmers, as there is less rainfall from July to September. Maroon farmers told us that low rainfall negatively affects rice yields, and therefore they sow rice at the beginning of the rainy season in December and in March / April. Introducing commercial wetland rice cultivars from coastal Suriname in Maroon communities should be done with care. Different environmental conditions in the interior such as soil type, rainfall, and bird predation are probably instrumental in the failure of the commercial cultivars that have been experimented with so far.

### Loss of seed stock

Seed dormancy and loss were a problem for Maroon farmers, as was shown by the three landraces that were too old to germinate. The most prominent reason for seed loss is poor storage facilities: the presence of moths, weevils, moisture, and rodents influences seed quality. Farmers said that rice must be sown within one year after harvest, because if the seeds are too old and if family members or friends do not have the same landrace in their field or in stock, such a variety might get lost. Farmers who cannot sow because of sickness, a temporary move to the city, or any other misfortunes risk losing all their crop seeds unless family, friends, or neighbors have the same landraces in stock. For these reasons, the recent initiative of the SNRI/ADRON and the Crop Trust to build community seed banks with improved storage facilities is greatly appreciated by the Maroon farmers (Crop Diversity Digest Staff 2024).

### Limitations of this research

Our research depended very much on the farmer’s schedule, making planning difficult. Farmers decided the sowing and harvesting dates, as well as what landrace to sow in which part of the field. We were limited to the landraces that farmers already had in stock and they were familiar with. Apart from the two commercial cultivars we did not introduce Maroon landraces that were unknown to the farmers. Therefore, we cannot compare the performance of all the Maroon rice landraces among the three locations. Our data also represent only one season (March-November 2023). Although it was an average year according to the farmers, we cannot predict the yields of these landraces in years with higher-than-average rainfall (such as in 2022) or exceptional droughts (such as in 2024). We also focused on a fraction of the hundreds of Maroon rice landraces. More research is needed on the relation between rice yield and soil fertility, rainfall variation, farm size, and socio-economic aspects that influence the amount of rice produced by Maroon farmers. The relationship between awns, hairy husks, and bird predation also requires further study. When different Maroon rice landraces are studied in the same location, by the same farmer, and with similar field preparations, clear differences between yield and growth duration may become visible.

## Conclusions

The discussion about traditional crop landraces and yield should consider under which conditions those landraces were originally selected and developed. If yield and maturation periods are measured under experimental settings the results should be interpreted carefully. The Maroon rice landraces we measured had a higher yield than suggested previously. Maroons never calculate their yield in kilogram per hectare, but they estimate how long a family can eat from their rice harvest. In general, we conclude that replacing Maroon landraces with commercial cultivars will most likely result in a decrease in rice yields and therefore lead to less food security. Also notable is that Maroon rice yield is comparable to that of rice landraces in Asia grown under low-input conditions.

## Supplementary Information

Below is the link to the electronic supplementary material.

Supplementary file1 (XLSX 27 kb)

Supplementary file2 (XLSX 28 kb)

Supplementary file3 (XLSX 14 kb)

## Data Availability

The authors confirms that the data supporting the findings of this study are available within the article and its supplementary materials.
